# Examining the emergence of digital society and the digital divide in India: A comparative evaluation between urban and rural areas

**DOI:** 10.3389/fsoc.2023.1145221

**Published:** 2023-04-12

**Authors:** Mahmudul Hasan Laskar

**Affiliations:** Department of Sociology, University of Science and Technology, Baridua, Meghalaya, India

**Keywords:** digital society, digital divide, digitalization in India, social transformation, digital inequality

## Abstract

Contemporary digital society has become a critical agent for transformation in various spheres of life and a new methodological framework for interdisciplinary research. It has emerged as a parallel entity alongside conventional society, where an individual's membership is not only limited to the physical world but also extends to the digital realm. In fact, a person's membership in the physical world is incomplete without their connection to the digital society. Digital technology is instrumental in driving social transformations in areas such as the economy, politics, culture, and religion. The striking feature of digital society is digital data production in the form of big data. Unlike in a conventional society, people's every move and behavior in a digital society are calculated and recorded as data. In this global context of a digital society, India has created opportunities for digitalization for its people since 2000, with significant strides made between 2015 and 2016. Reliance Jio, a telecom company, helped to accelerate this process by offering free unlimited Internet packages on a mass scale. This led to a tremendous surge in service industries and the emergence of new sectors, as well as a digital revolution in the conventional systems of the economy, politics, culture, education, religion, and law. However, this transformation has also exposed a significant challenge—the digital divide or digital inequalities, which cannot be overlooked or undermined in sociological research. It would be wrong to reduce digital inequality to a mere technological divide; it is a complex issue shaped by prevailing socioeconomic conditions, digital inequalities, and capability inequality. The study revealed that India's prevailing socioeconomic divide is the source of its wide digital divide. This digital divide exists across both rural and urban areas, affecting access to digital education and economic opportunities. The digital divide is also found between under-resourced urban areas and affluent residential areas. This study's theoretical framework draws on the studies of Castell on the information society and Dijk's concept of the network society.

## Introduction

The growing global socioeconomic divide and the digital divide are currently the most pressing issues that sociology is grappling with. The world is undergoing a digital transformation. As a result, digital society, digital space, digital capital, digital relations, digital access, and so on have become increasingly relevant to sociology, like any other societal aspect and social relations. The digitalized world is described using various terms, such as information society (Dijk, 2006) and network society (Dijk, 2006; Castells, 2010). These terms are used to denote the society that has emerged from the impact of information and communication technology. The most commonly used term is information society. Castells (2010) and Dijk (2006) have used the terms “information society” and “network society” to describe this type of society.

In the 1970s, the information and communication technological paradigm in the United States initiated a technological development that resulted in the digital revolution. This new paradigm shifted European and Western societies' political and economic conditions. The new technological paradigm surpassed the early rise of the electronics industry in the 1940s−1960s, as it fostered a culture of freedom, individual innovation, and entrepreneurialism (Castells, 2010, p. 4). An important question to consider is whether technology has developed uniformly on a global scale. Did technology determine different levels of development? Did society determine different levels of technological advancement? The digital divide question makes us consider societal or state intervention in technological advancement and vice versa. Castells (2010, p. 5) believes that technology does not determine society but society does determine the course of technological change because many factors, such as individual inventiveness and entrepreneurialism, are involved in the process of scientific discovery, technological innovation, and social applications. However, the results depend on the complex interaction pattern between technology and society. He further argues that technological determinism is a false problem because technology is society, and society cannot be understood or represented without its technological tools.

Society and technology are inevitably linked, and society determines technology and its advancement. Digital society can be viewed as a parallel to physical society, in which individuals voluntarily become members. However, its membership is as important as the membership of the physical society. Physical society refers to the material existence of social institutions and the physical appearance of social relations. We accept that abstract entities, such as norms, values, and customs, are essential societal elements. In the physical society, human actions and relationships are regulated socially, culturally, and legally, while in the digital society, online behavior is governed by simple legal guidelines. However, these guidelines cannot be equated with social control mechanisms present in physical society.

A question then arises: Does society determine the digital divide? The answer to this question is yes, because the prevailing socioeconomic divide is the root cause of the digital divide in any society. In this context, it can be argued that the digital divide cannot be understood in isolation but rather in relation to the global socioeconomic divide. India has been part of the digital society since the beginning of the 21st century with the introduction of the Internet and information and communication technology. However, it was not until a significant shift occurred in 2016 with the launch of Reliance Jio Infocomm Ltd. by Reliance Communication Ltd. that a true digital revolution took hold. This revolution marked the beginning of a new age of digitalization in India, leading to a subsequent smartphone revolution.

## Materials and methods

The study is based on qualitative methods such as unstructured interviews, case studies, and participant observation. The present study conducted a review and interpretation of published studies and also included an empirical study. The study selected Guwahati, the capital city of Assam, and Buribail village (District Cachar) in Assam as its locations. The respondents were students, working youths, professionals, and rural dwellers. In the unstructured group interview, 80 interviews were conducted in both villages and cities. Case studies were conducted in under-resourced areas in Guwahati city where the living conditions of some houses and dwellers were analyzed. Additionally, participant observation methods were used among students and youths in urban residential areas, such as apartments. The study, which focused on a city and a village, can serve as a hypothesis for further research on the issue of the digital divide. Moreover, this study mainly focused on interpreting the inevitable link between socioeconomic disparity and the digital divide in India. The theoretical framework of this study highlights the interconnectedness of digital society and physical society, emphasizing that the digital divide cannot be reduced to a mere technological problem but must be understood as a complex techno-social problem.

## Results

The results of the study revealed that digital society is a new techno-social phase that has emerged globally, and India has become a part of it. However, India has not been able to address the issue of socioeconomic inequality. India's prevailing socioeconomic divide is the source of a wide digital divide. The digital divide was found to exist primarily in the educational and economic aspects of both rural and urban areas. There is a wide digital divide between rural and urban areas, as well as between affluent cities and under-resourced urban areas. The digital divide mainly includes poor digital infrastructure in villages and under-resourced urban areas, limited access to digital facilities, and poor socioeconomic conditions. However, cities and affluent parts of cities are technologically advanced and have access to digital facilities. A detailed discussion is made in the sections below.

### Digital society: A new techno-social phase

The first question that arises is as follows: Do we differentiate digital society from network society or information society? Our approach was not to consider the digital society as a distinct entity but rather as an extension of the network society or information society. An important issue that has been highlighted is how digital society encompasses societal dimensions and turns into a parallel society. Instead of focusing on defining the digital society as a separate physical entity, it may be more useful to revisit Castell's (2010) concept of the network society. He elaborated on how the emergence of a new technology or technological paradigm, based on the development of information and communication technology, has transformed the world since 1970 and diffused unevenly across various regions of the world. Technology does not determine society; rather, it is a society that determines technology. This indicates that technology is regulated by the values, norms, and needs of people within society, as stated by Castells (2010). Castells (2010) coined the term “network society” and referred to the emerging society as a network society. He preferred this term over information society or knowledge society because microelectronics-based networking technologies brought new capabilities to an old form of social organization in the knowledge society or the information society. He claimed that digital communication networks are the backbone of the network society (Castells, 2005, p. 4). The network society is based on communication networks that transcend boundaries, making the network society global. The network society is based on global networks of capital, goods, services, labor, communication, information, science, and technology. Due to their programming, networks are selective, and as a result, while the contemporary network society has diffused throughout the world, it still does not include everyone. Although global networks have an impact on all human beings, they have excluded large sections of the population in the early 21st century (Castells, 2005, p. 5). Dijk's (2006) concept of information society and network society signifies the contemporary development of modern society, characterized by widespread information exchange and the use of information and communication technology in every sphere of life. His classification of society as an information and network society also corresponds with concepts such as capitalist society and post-modern society. Information is a fundamental aspect of an information society that permeates all aspects of society. In an information society, the societal organization is based on science, rationality, and reflexivity; the economy (agrarian and industrial) increasingly leads toward information production, and the labor market is based on information processing skills that require knowledge and education. Culture in the information society is determined by media and information products through various symbolic entities and meanings (Dijk, 2006, p. 19). The network society is defined as a social structure that relies on social and media networks as its infrastructure, with organizational networks existing at the individual, group, or organizational and societal levels. The network function serves as a means of linking all units, including individuals, groups, and organizations. Individuals are the fundamental unit of a network society in the West, whereas families, communities, and workgroups may be the network units in Eastern societies (Dijk, 2006, p. 20). A network is defined as a collection of links between unit elements, where nodes represent the elements and the unit systems. A single link between two elements is called a relation(ship). Networks are a mode of organization for complex systems in nature and society (Dijk, 2006, p. 24), with the individual as the basic unit of a network society. Traditional collectivities such as joint families, communities, and tribes have become fragmented. Different kinds of communities have emerged that are connected through larger-scale networks, even though they continue to live in their traditional families, neighborhoods, and organizations. This development has made the work environment more extensive and connected due to the networks (Dijk, 2006, pp. 35–36).

Tim Berners-Lee's leading innovation, the World Wide Web, came along in 1989 and played a critical role in the digitalization of the world, which transformed Internet technology from a small network of computers into global communication systems. Until then, the Internet was used only for military and research purposes, but the World Wide Web broadened its network. With this technological transformation, the material foundation for a new society was laid: a digital society (Redshaw, 2020). Information flow is the key feature or basis of a digital society. Digital society and the information age have a definite pattern of social relations and communication (fundamental structural elements of society) derived from a complex system of relations in the physical society. On the one hand, the term “physical society” refers to a society where people live in systems of physical contact and have face-to-face relationships. On the other hand, digital society is a virtual system of social relations that allows individuals to gain membership and citizenship through the use of the World Wide Web and the Internet. The establishment and operation of a physical society require physical infrastructure and an environment. Similarly, the establishment and operation of a digital society require digital infrastructure such as computers, mobile phones, the World Wide Web, the Internet, and applications.

The digital society is characterized by a flow of information through global networks at unprecedented speeds. The “superconnected” life through the “Internet of things” is the most striking feature of digital society, where big data and data mining play a crucial role (Redshaw, 2020). The digital society became a global transformation due to the fourth industrial revolution, known as Industry 4.0. This techno-social transformation is closely linked with consumerism, and it aligns with the current advanced industrial age for greater market benefits and industrial benefits. Late capitalism or advanced capitalism extensively regulates society, political economy, and everyday life to the extent that all human activities are digitalized and become objects of profit for industries. The digitalization of life has undoubtedly upgraded the standard of living, but, at the same time, it has raised many questions regarding digital use and consumption, digital trends or commercial trends, power games, and so on. The consumption trend, particularly in popular culture and social media in India, has been growing fast, raising questions about digital space, the capitalization of digital space, digital activism, and the digital divide. The culture industry or popular culture (Horkheimer and Adorno, 1944; Marcuse, 1964) is legitimately utilized or rationalized with the notion of “entertainment” in an advanced industrial society. In contemporary society, social media is not just a medium of communication but rather an industry that generates its audience and produces content. Social media has recently become a new reality and a digital phenomenon. Industry 4.0 marked the beginning of a new digital society where people's access to technological infrastructure and the Internet is inevitable. Industry 4.0 refers to a digital, data-driven, interconnected industry that transforms production, marketing, labor, healthcare, and human relations, among others (Banholzer, 2022). Those who cannot access technological infrastructure and the Internet are cut off from mainstream society and the global village. From the point of access and capability of people, digital inequality and the digital divide appear as major global phenomena. The European Commission (2021) planned a highly innovative and ambitious industrial strategy for Europe. The main focus was on creating an economic transformation in Europe to augment the change. Industry 4.0 was coined in Germany in 2011 as a future project of the country and an integral element of its high-tech industrial strategy. It mainly focused on the stability of employees in production systems and ecological dimensions in the form of “green production” for a carbon-neutral and energy-efficient industry (European Commission, 2021, p. 8). The network society is instrumental in developing the digital society. The evolution from a network society to a digital society is referenced in Dijk's (2006) analysis of the development of information and communication technology. The most striking foundation of a network society is microelectronic technology, which enables telecommunications, data communications, and mass communications to be carried out. This technology is the basis for the improvement of communication capacities in the new media, including speed, storage capacity, accuracy, stimulus richness, and complexity of operations. The second fundamental structure of network society is the use of a uniform language in microelectronics for exchanging signals. This uniformity is the language of digital signals. Digitalization is the binding structure for all new telecommunication, data, and mass media networks (Dijk, 2006, p. 43). Digitalization refers to the process of converting analog signals, such as sounds and images, into digital signals made up of ones and zeroes (bits). This allows for the fast and uninterrupted transmission and connection of these signals with the help of microelectronics. Digital signals can be easily processed and manipulated to improve the quality of data, texts, sounds, and images (Dijk, 2006, p. 44).

The digitalization of the network society is a new technological development in the world's communication system. Questions that arise are as follows: Is it only about communication or more than this? To take a critical theory, or the Frankfurt School's stance of “techno-rationality” and “democratic unfreedom,” we can claim that, although the technology of communication and networking is created by the subjective state of human beings, it has been determined by the objective social structural mechanisms of society. Objective social structural elements may be industry, statism, economic policy, the political power system, and so on. Therefore, communication in a network society or information society is not simply a mechanism of human relations and exchanges but rather a source of a new mode of control. Techno-rationality legitimizes democratic freedom by creating the mechanisms (digital nods) of a lack of freedom in an industrial system. Humans use digital technology that is already programmed in such a way that its result will meet the desired goal of the industry.

The digital society and physical society cannot function separately because elements such as social relationships, societal structure, social institutions, norms, values, culture, and social control are redefined as complex techno-social systems in the new digital world. This technocratic social structure of the digital society has significantly altered the traditional activities of the physical society. The digital society represents a blend of techno-social and economic progress. This advancement is what neo-Marxists like Adorno and Horkheimer (1944), Marcuse (1964), and Ralf (1957) called an advanced industrial society. Membership in a digital society is not mandatory, and individuals having the option to choose is just as crucial as being a member of a physical society. However, its membership is as important as the membership of the physical society. This is because the vast influence and widespread integration of digital technologies into human life make it impossible for any individual worldwide to remain isolated from the digital society. In short, the term “physical society” refers to the tangible presence of social institutions and the observable connections within social relationships. However, it is important to recognize that abstract entities such as norms, values, customs, and so on are also inevitable elements of society that have no physical existence but appear in the social activities of human beings.

### Conceptualizing the digital divide in the Indian context

**Figure 1:**
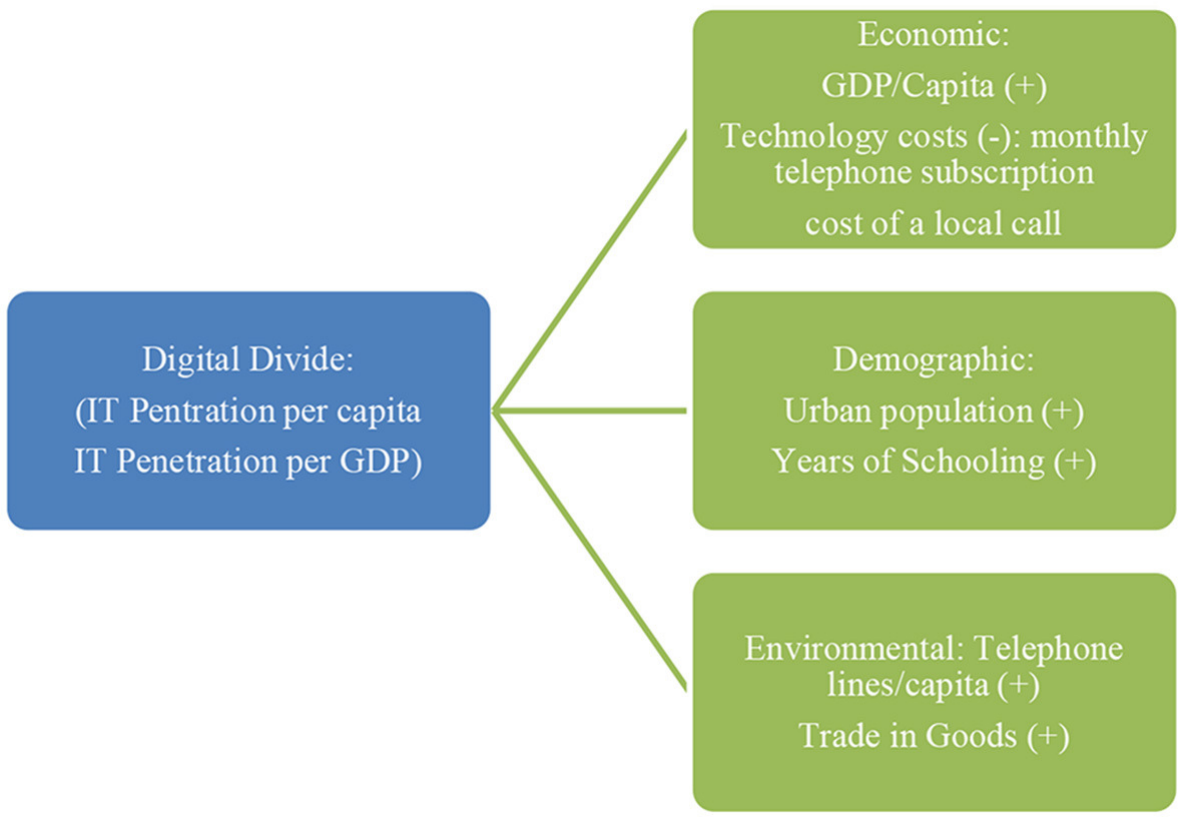
An empirical framework for examining the digital divide. Source: Kraemer et al. (2005), p. 414.

Just as the globalization of technology and digital society reshaped the world, the digital divide added a new dimension to the persisting global socioeconomic divide. Addressing the digital divide has become a global concern due to the significant role that technological progress and techno-consumption play in the global political economy (Figure 1).

Bridging the digital divide is beneficial for businesses because the marketplace is online, and, in this case, having more online customers is profitable (Riggins and Dewan, 2005). There are three levels of analysis of the policy and managerial implications of the digital divide (Riggins and Dewan, 2005, p. 300).

Individual level: At the individual level, the “digital divide” refers to a lack of access to IT due to technological, sociological, and economic disadvantages. The gap exists between individuals who have access to IT as an integral part of their lives and those who do not. Access to technology also varies across geographical areas. For instance, rural areas have poor access to the Internet (Riggins and Dewan, 2005, p. 300).

**Organizational level:** At the organizational level, the “digital divide” refers to the disparity in digital management among industry organizations.

**Global level:** At the global level, the “digital divide” refers to the inequality in investment and policies for both corporate and individual adoption of ICT among different countries (Riggins and Dewan, 2005, p. 300).

The analysis of the digital divide at these three levels considers two types of effects: first-order effects, which relate to inequalities in access to ICT, and second-order effects, which relate to inequalities in the ability to effectively utilize ICT, among those who already have access (Riggins and Dewan, 2005, p. 300).

The digital divide refers to the gap between individuals, households, businesses, and geographic areas at different socioeconomic levels regarding opportunities to access information and communication technologies (ICTs) and their use of the Internet for a wide variety of activities. However, access to telecommunications is a precondition for the access and use of the Internet (OECD India, 2021, p. 5).

The pattern of the digital divide in India is found at three levels:

Social group or class (lower caste and lower class): The digital divide between higher and lower castes is a socio-technical issue that highlights how higher castes tend to be more affluent and digitally advanced than lower castes. The class or socioeconomic gap is vast in Indian society; therefore, the digital divide is a kind of extension of the prevailing divide (Understanding the Digital Divide, 2001).

**Individual**: The digital divide is determined by an individual's ability to access digital resources, their level of digital literacy, their reasons for using digital platforms, and their consumption habits.

- **Institutions** (education, governance, and local economy): At the institutional level, the digital divide can be observed in three areas: public and private schools, colleges, and universities. The digital divide in governance refers to the divide between local self-governance (Panchayati Raj Institution) and people's access to digital governance. The digital divide is also visibly prevalent in the local economy.

### The interface of the socioeconomic and digital divide in India

**Figure 2:**
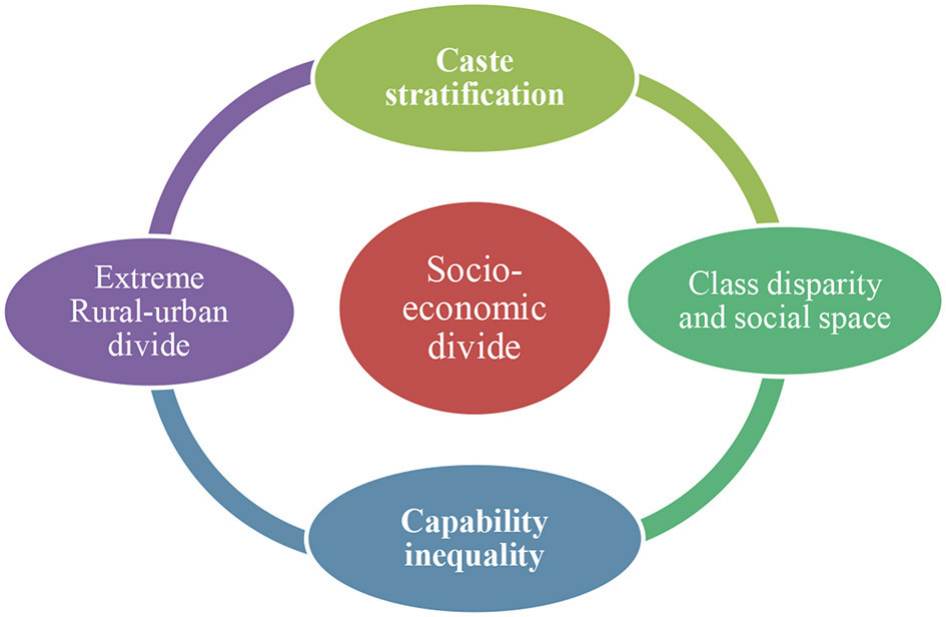
The interface of the socioeconomic and digital divides in India. Source: A field study in village and city in Assam, India.

The digital divide in India cannot be analyzed as a single issue. Considering that pre-existing socioeconomic divides is also important, India is characterized by various socioeconomic divides, such as caste stratification, the rural-urban divide, capability inequality, and class disparity. Dalits are considered to be the lowest stratum of caste groups in India, whose social and economic position is much worse than that of any other population in India. India's rural areas are still highly under-resourced and poorly managed compared to its urban areas, which are much more developed and technologically advanced (Figure 2).

Capability inequality is another serious issue to consider (Sen, 1993, 2009). India exhibits various patterns of living standards based on capability. The class gap and social stratification in India are concerning, as there is a clear divide between the affluent and poorer sections of society, both in rural and urban areas.

### Fields of the digital divide in India

**Economic:** The economic situations of individuals and social groups are based on their occupations. In any society, people maintain two kinds of economic bases: job/work and skills. India has a serious issue with work opportunities due to a lack of skills or poor technical skills. In contemporary society, static skills are no longer relevant; individuals need to continuously upskill themselves to survive in the competitive market. Unfortunately, the low attainment of education is a problem, resulting in a limited number of technically skilled laborers. Therefore, digitalization may not be able to bring about significant changes in the working conditions and overall economy of manual workers (India Skills Report, 2021).

India's employment trend is largely informal, with a large percentage of the workforce engaged in informal jobs. This informal workforce in India encompasses individuals working in private enterprises, daily wage laborers, domestic helpers, and manual laborers in the formal sector who work without any socioeconomic security or benefits. The total population employed in India is 461.52 million, of which 415.23 million have informal jobs. Of the total number of people employed, 90% of men and 92% of women are informally employed. The education of workers in the informal sector is low. Educational attainment is also low among domestic laborers, street vendors, sweepers, and manual construction workers. Of these workers, over 60% of women and 35% of men had dropped out of primary school (World Bank Report).

**Education:** The digital divide is also evident in certain aspects of education.

Institutional Difference: This is the difference between public and private educational institutions in digitalized learning.

Unequal access to digital infrastructure: Affluent sections have the necessary digital equipment to pursue education, but poorer sections are unable to take advantage of these resources.

Unequal access to e-learning: E-learning platforms, such as BYJUS, Unacademy, and WhiteHat JN., have become very popular in India. However, due to unequal access to digital infrastructure, not all sections of the population have equal opportunities to benefit from these platforms.

**Figure 3:**
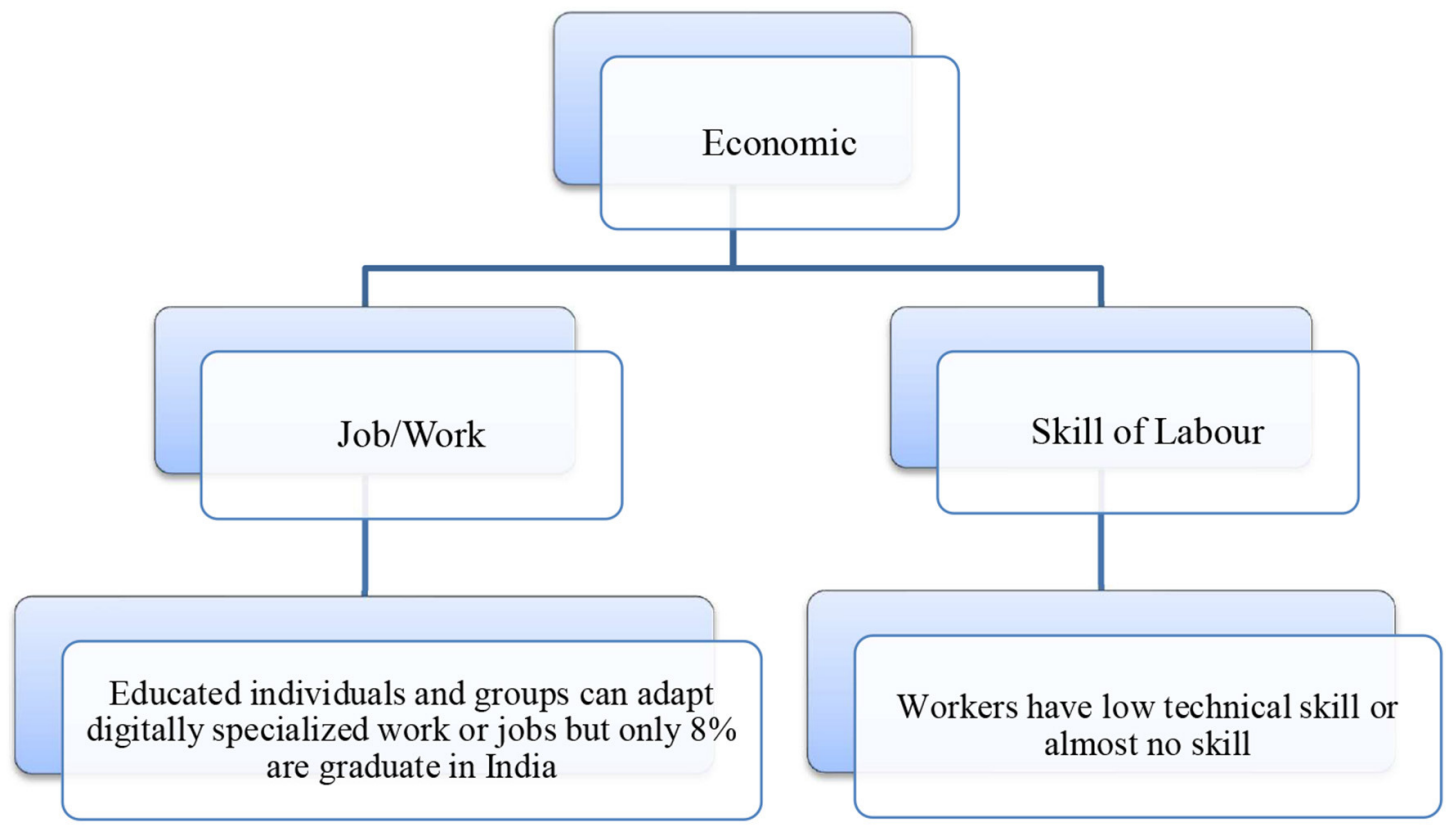
Economic dimension of the digital divide in India. Source: Census India (2011) & field study conducted in village and city in Assam.

The social environment for digitalized education: The social environment in under-resourced villages and under-resourced urban areas is not conducive enough for education even if mobile phones are available; the quality of education remains poor (Figure 3).

### Digitalization and the trend of popular culture consumption in India

In 2007, Reliance Jio sparked a revolution in India by providing unlimited 3G and 4G Internet access at an affordable price. This Indian telecommunications company, also known as Jio, launched its commercial 4G services in 2016 with free data and voice services, a strategy that was later adopted by other telecom companies such as Airtel, BSNL, and more. Moreover, the partner policy of mobile phone companies that began offering 4G smartphones at much lower prices also played a significant role in contributing to the growth of Jio, Airtel, BSNL, etc. The availability of smartphones from Chinese mobile companies in the Indian market changed the entire consumption pattern. Compared to Nokia and Samsung, Chinese companies (Oppo, Vivo, Huawei, Karbonn, etc.) offered smartphones at a significantly lower price, which was easily affordable for most people in India. Later, Nokia and Samsung also followed the same policy to create a greater customer network. These Chinese smartphone companies ensured the purchasing capacity of mobile phones for workers such as rickshaw pullers, daily wage laborers, and factory workers. Thus, Reliance Jio's data plans and a new policy among smartphone companies, particularly Chinese companies, contributed to the advancement of consumer networks that eventually helped popular culture and social networking to grow exponentially. The social network transformed after the advent of Web 2.0 in the early 21st century, evolving into comprehensive platforms for data collection, analysis, and content creation. The integration of social networks with popular culture has been instrumental in the massive growth of the digital population, ultimately positioning India as a leading consumer trendsetter in the world.

Upon examining the widespread use of social media, it becomes apparent that it functions as both a component of the culture industry and a commercial media industry. O'Reilly and Battelle (2009) concept of Web 2.0 has shed light on the evolution from “network as service” to “network as platform.” “Network as platform” refers to harnessing the power of networks to create applications, which is key to attracting and engaging users. The industry encompasses huge organizations that facilitate software provision and a community of users connected by social networking platforms such as Facebook, YouTube, etc. Mandiberg's (2012) concepts of “amateur media” and “user-generated content” showed us how social media had enabled user participation in media in two ways: as both producers and consumers. Media consumption has changed from a unidirectional to a multidirectional system. Mandiberg (2012) noted the massive growth of social media use because of the affordability of computers, software, and the Internet in the early 21st century. Data scientists have claimed that “media analytics” (Manovich, 2018) is the basis for the consistent growth of the culture industry. Social media has changed the prevailing media system from a unidirectional media-audience relationship to a multidirectional communication process. People are presently not merely passive audiences but active participants; technological advancement has enabled the majority of people to use social media to produce and distribute content. Until the end of the 20th century, media was a professional organization, and people were simply audience members. However, this relationship has become less distinct because of the multidirectional broadcasting started by new media forms such as blogs and social networking sites, which focus on active audience participation instead of unidirectional broadcasting. This happened mainly because of the affordability of computers, software, and the Internet; the majority of people were able to purchase these for personal use (Mandiberg, 2012, p. 1). Significant technological innovations in social media have led to the development of participatory systems such as messaging applications, like and dislike buttons, sharing options for various types of content (photos, videos, blogs, and other contents), and comment features. Mandiberg called these types of social media content “user-generated content” (Mandiberg, 2012, p. 2). It is interesting to note that technological intelligence creates a huge community of users of applications and produces user-generated data in real time, which is useful for social networks, culture industries, and business platforms such as Amazon, Google, and so on. Seeing the massive change in network applications (software), Tim O'Reilly (2012) conceptualized Web 2.0 to describe the network as a platform for user-generated and industrial use of real-time user-generated data. Web 2.0 harnesses collective intelligence and can be considered a mature stage of the web (formally known as the World Wide Web) (O'Reilly and Battelle, 2009). Network applications are systems for harnessing collective intelligence that depends on managing, understanding, and responding to massive amounts of user-generated data in real time, which include data subsystems such as location and identity (of people, products, and places). The smartphone revolution further widened the web as it moved from desktops to people's pockets. The most remarkable addition to Web 2.0 is the use of sensors in web searches instead of manual human searches; motion and location sensors extensively record human activity and store it as data. In this day and age, people's every activity, including their choices of food, clothing, and places; their engagement with popular culture items; their time spent using social media; the nature of the content they follow; and their current locations or previously visited locations, are recorded as data (big data) in the era of advanced Web 2.0. The culture industry, social media, and other service industries collect, present, and use this data in real time (O'Reilly and Battelle, 2009, p. 1).

Real-time media analytics represents a major technological breakthrough in service industries. Companies sell cultural goods and services through websites and apps (such as Amazon, Apple, Spotify, and Netflix) that organize searchable information for their users. This is made possible through platforms such as Google, Baidu, and Yandex. To increase sales and attract more consumers, these companies utilize social communication and information-sharing tools such as Facebook, QQ, WeChat, WhatsApp, and Twitter. Additionally, they use media-sharing platforms such as Instagram, Pinterest, YouTube, and iQiyi. These companies rely on the computational analysis of massive media data sets and data streams. The practice of analyzing large amounts of content and interaction data across the culture industry began in 1995 and matured in 2010 when Facebook reached 500 million users. These data include information on users' online behavior, physical activity, media content created by companies, and media content created by users of social networks. People's online behavior is monitored through browsing pages, link tracking, post sharing, post linking, content viewing, content playing or reading, and ad clicking. Physical activity data pertain to social network and online platform usage, including the time and location of use. Media content created by companies are, for example, songs, movies, videos, and books; and media content created by users of social networks includes posts, conversations, images, and videos (Manovich, 2018). Companies have used two kinds of data: “data sets” (static or historical data) and “data streams” (data in real-time). However, currently, industries are increasingly using real-time data analysis. Sociology, digital humanities, and computational social sciences analyze data sets or historical data.

When widespread poverty, hunger, unemployment, and poor living conditions are prevalent, the apparent poor status of social wellbeing in India becomes the topic of discussion. As such, the consumption of popular culture and social media in India may not be entirely justifiable, as leisure and entertainment are often reserved for industrial workers or employees in the United States and other advanced industrial societies (started by Fordism), who have the means and material wellbeing to consume popular culture for relaxation. Therefore, the consumption of popular culture and the notions of “entertainment” and “free time” are linked (Horkheimer and Adorno, 2002), which is the basis of labor management for mass production (Fordism) in an advanced industrial society. In India, a large number of people are unemployed and struggle to maintain a basic standard of living and access to education and healthcare.

The Oxfam International (2021) revealed that the top 10% of the Indian population holds 77% of the total national wealth, indicating a vast class gap and extreme inequality. Therefore, mere consumption growth does not necessarily validate progress in a nation like India. Despite these challenges, India had a digital population of 468 million in 2020, which has been continuously growing due to the country's large population. The culture industry has benefited from this growth, as popular culture production and consumption are widespread. The entertainment business in India has thus been growing rapidly, albeit against a backdrop of inequality and socioeconomic challenges. According to the FICCI and EY (2020, 2021) report on Media and Entertainment in India, Indians spent 4.5 h a day on their phones in 2020, a significant increase from 3.5 h in 2019 and a 25% increase from 2017. With 4.5 h per day, India held the 3rd position in the world for the most time spent on phones in 2020, surpassing China, Mexico, Argentina, and South Korea. Additionally, consumers in India spent 1,669 billion minutes online in 2020, a 32% increase compared to 1,261 billion minutes in 2019.

### The digital divide in terms of geographical areas in India

#### Rural–urban digital divide

The digital inclusion policy aims to expand the reach of the digital network to cover more areas. Given that the rural population constitutes nearly 70% of India's total population, including rural areas in the digital network would benefit the telecom and service industries. However, there is currently a lack of initiatives to enable rural people to adopt digital facilities. What can we teach a rural person with low literacy skills about technology? Will he/she be able to use Google or any app? In the survey, it was found that rural areas have inadequate digital infrastructure, limited access to the digital world, and insufficient capability to make use of digital facilities. Although Internet connections are available in villages and smartphones are available to almost everyone, they are mostly used for making phone calls and consuming entertainment content.

A total of 50 urban respondents and 30 rural respondents participated in the study. Of the 50 urban respondents, 30 were from under-resourced urban areas. A set of questions related to education and the economy were used in the study, with a focus on unstructured group interviews and observation to obtain a better understanding of the issue. The data were presented in a qualitative form, as the study did not use statistical analysis. The digital divide between rural and urban areas was found to exist mainly in two aspects: education and digital economy.

***Education:*** Rural youths were found to be fascinated by the world of new media and popular culture. However, their usage patterns were more for entertainment than for productive, learning-oriented, or skill-oriented purposes. The schools in the surveyed village were found to be functioning with minimum facilities and without any digital infrastructure. The teachers were not well-versed in technology related to teaching and learning. The absence of colleges and vocational and training institutions in villages is another major issue that hinders the participation of villages in global digitalization. In contrast, urban schools and colleges have better access to digital technology, and teachers are well acquainted with the use of technology. An interesting fact is that urban children use facilities such as e-learning and other digital learning platforms such as BYJU's, Unacademy, WhiteHat Jr., and so on, while rural children are unable to access such facilities mainly due to a lack of proficiency in using technology, poor digital infrastructure, and a lack of resources.

***Digital economy:*** The poor condition of digital agriculture and the lack of access of rural dwellers to digital agriculture are the main causes of the divide between rural and urban areas. The economy in urban areas is relatively more developed at the micro level. In contemporary society, small businesses are regulated on digital platforms, and digitalized service industries have transformed people's economic situations. Almost the entire rural economy in India is dependent on agriculture; therefore, its digitalization is inevitable, but it has been found that villagers are still unaware of digital agriculture. There is no evidence of digitalized agriculture, so rural dwellers' access to it is still a distant dream. Villagers hardly keep track of the market price of their products; they sell them to middlemen or traders in the local market at very low prices. Villagers have no knowledge of local branding of their products, marketing on digital platforms, or online business; they conduct payment through Google Pay/Phone Pay. The irony of the situation is that rural dwellers sell their agricultural products to traders who take them to nearby towns and cities. The same products are purchased by villagers working in towns at a price much higher than the price his/her fellow village dwellers levied.

#### Under-resourced urban areas and urban areas

Social stratification and the digital divide in the urban areas of India are evident. There is a wide distinction between city dwellers (the sophisticated urban population) and dwellers of under-resourced urban areas. The population in under-resourced urban areas has been increasing in India, with poor quality of life, limited digital access, and inadequate digital life facilities. As of 2022, the number of people living in under-resourced urban areas reached ~100 million, which is greater than the entire population of Australia (India Housing Report, 2022). Under-resourced areas in Indian cities are widespread and attract a large population working in the unorganized sector, including those who are unemployed, homeless migrants, daily wage laborers, baggers, and vendors. We found a wide gap between the affluent residential areas of the city and under-resourced urban areas in terms of facilities, living conditions, and standards of living. People living in under-resourced urban areas are very poor. Although digitalization has penetrated under-resourced urban areas, it has not brought significant changes because people are already vulnerable in many social and economic aspects. People who live in under-resourced urban areas do not have access to digitalized education, e-health care, the digital economy, or technology-based skills. They own smartphones, but the study revealed that they mostly use them to consume popular culture and social media. Even school-going children are often found loitering and using mobile phones for various non-social and unethical activities.

Their counterparts in the affluent residential areas of the city are enrolled in prestigious schools such as international schools, convent schools, and residential schools, among others. These schools are equipped with advanced digital technology, and students have no issues accessing digital facilities. However, children living in under-resourced urban areas face difficulties accessing digital facilities. The schools in which children from under-resourced urban areas are enrolled have inadequate physical infrastructure and digital infrastructure, and the academic environment is so poor that children often come only for midday meals (a government scheme for providing lunch to children). Most people who live in under-resourced urban areas have low literacy skills and lack any basic knowledge of the digital economy or technical skills. Their living conditions are so impoverished that socioeconomic development should be prioritized over digitalization. They prioritize access to drinking water, personal toilets, a hygienic house, and sanitation. We found that almost all households in under-resourced urban areas have access to television and smartphones, but this does not equate to digitalization. Under-resourced urban areas have been included in the digital market as consumers, but digitalization has not acted as an agent of socioeconomic transformation. Manual laborers with education up to at least secondary and senior secondary are switching to technical fields and undertaking training programs to upgrade their skills. However, in under-resourced urban areas, the highest level of education attained is often only up to the 12th grade. Most residents have either no literacy skills or have a limited education that barely allows them to write their own names. This significant digital divide between affluent city dwellers and those who live in under-resourced urban areas has made the challenge of creating a smart city even more difficult and uneven. Although a city may be considered smart, because digitalization has not been able to transform the entire urban society, it could be argued that a smart city lacks a smart society.

## Conclusion

India's digital divide is not just a technological challenge but a reflection of the country's longstanding socioeconomic disparities. Digital inclusion efforts must focus on developing the capabilities of all sections of society rather than simply expanding digital infrastructure. This requires a concentrated effort to improve educational attainment, socioeconomic status, and digital literacy. Without these efforts, the benefits of digitalization will not be fully realized. It is important to recognize that a smart city cannot exist in isolation from broader society, and efforts must be made to address the realities of under-resourced urban areas and other marginalized communities. Ultimately, the goal should be to create a smart society in line with the concept of Society 5.0, where technology is used to enhance the wellbeing of all members of society.

## Data availability statement

The original contributions presented in the study are included in the article/supplementary material, further inquiries can be directed to the corresponding author.

## Ethics statement

Ethical approval was not required for the study on human participants in accordance with the local legislation and institutional requirements, as confirmed by the Sociology Department Research Committee of the University of Science and Technology, Meghalaya. The participants provided their written informed consent to participate in this study.

## Author contributions

The author confirms being the sole contributor of this work and has approved it for publication.
